# Why are mini-implants lost: The value of the implantation technique!

**DOI:** 10.1590/2176-9451.20.1.023-029.oin

**Published:** 2015

**Authors:** Fabio Lourenço Romano, Alberto Consolaro

**Affiliations:** 1 Professor, FORP-USP; 2 Full professor, School of Dentistry - University of São Paulo/Bauru

**Keywords:** Mini-implants, Orthodontic anchorage, Bone screws, Corrective Orthodontics

## Abstract

The use of mini-implants have made a major contribution to orthodontic treatment. Demand has aroused scientific curiosity about implant placement procedures and techniques. However, the reasons for instability have not yet been made totally clear. The aim of this article is to establish a relationship between implant placement technique and mini-implant success rates by means of examining the following hypotheses: 1) Sites of poor alveolar bone and little space between roots lead to inadequate implant placement; 2) Different sites require mini-implants of different sizes! Implant size should respect alveolar bone diameter; 3) Properly determining mini-implant placement site provides ease for implant placement and contributes to stability; 4) The more precise the lancing procedures, the better the implant placement technique; 5) Self-drilling does not mean higher pressures; 6) Knowing where implant placement should end decreases the risk of complications and mini-implant loss.

## Abstract (pt)

O uso de mini-implantes trouxe grandes contribuições ao tratamento ortodôntico. Essa demanda gerou curiosidade científica sobre os procedimentos e técnicas de implantação. Entretanto, instabilidades desses dispositivos ocorrem por motivos ainda não totalmente esclarecidos. Objetiva-se, com esse trabalho, relacionar a técnica de implantação com a taxa de sucesso dos mini-implantes por meio das seguintes hipóteses: 1) áreas com osso alveolar pobre e com pouco espaço inter-radicular levam à inadequada implantação; 2) diferentes áreas requerem distintos tamanhos de mini-implantes! O tamanho do implante deve acompanhar o diâmetro do osso alveolar; 3) a correta determinação do local em que será colocado o mini-implante facilita a instalação e contribui para a estabilidade; 4) quanto mais precisa for a lancetagem, melhor será a técnica de implantação; 5) autoperfuração não significa alta pressão; 6) saber onde finalizar a implantação diminui a incidência de complicações e de perda dos mini-implantes.

## Proper technique, greater chances of mini-implant placement success!

There certainly was high-quality Orthodontics before the advent of mini-implants. Severe malocclusions were treated and, by the end of treatment, the ideal objectives of orthodontic therapy were achieved. Professional skills and clinical experience in similar cases contributed to establish a stable and functional occlusion. Nevertheless, more complex orthodontic mechanics occasionally led to or allowed unwanted movements of teeth involved in appliance use. Thus, there was a need to control such side effects so as to allow treatment to be properly developed.

Once mini-implants were introduced with a view to aiding orthodontic treatment, they allowed unwanted effects to be minimized or even eliminated, thereby favoring tooth movement mechanical control. This resource caused major changes in current orthodontic treatment.

Implant placement technique is relatively simple and does not require an oral and maxillofacial surgeon. Mini-implants can be installed by an orthodontist, provided that previous planning has been made with a proper sequence of procedures that respect all clinical steps. In addition, patient's anatomical features should be carefully considered together with the limitations imposed by the technique. 

Initially, patients should be advised of the risks and benefits provided by mini-implants. After having the patient's consent, planning should be carried out in accordance with the mechanics and clinical possibilities. Faced with any impossibilities, implant placement must be interrupted and a new planning should be done. Implant placement clinical sequence involves selecting a specific type of mini-implant according to the site in the oral cavity, taking radiographic examinations of the placement site, preparing the operating field through full asepsis, encouraging the patient to perform mouth washes with 0.12% chlorhexidine, performing anesthesia (topical and infiltration) and lancing procedures, carrying out mini-implant placement procedures and new radiographic examinations so as to check mini-implant positioning.

The literature^1^^-^ 9 ^,^ 10 ^,^ 12 ^,^ 13 ^,^ 14 provides numerous reports on orthodontic mini-implant placement procedures; however, little attention has been given to why it should be done so. There is plenty of research and reflection to be done.

## Hypotheses to explain mini-implant loss in absolute orthodontic anchorage

## 1) Sites of poor alveolar bone and little space between roots lead to inadequate implant placement!

**Figure 1 -:**
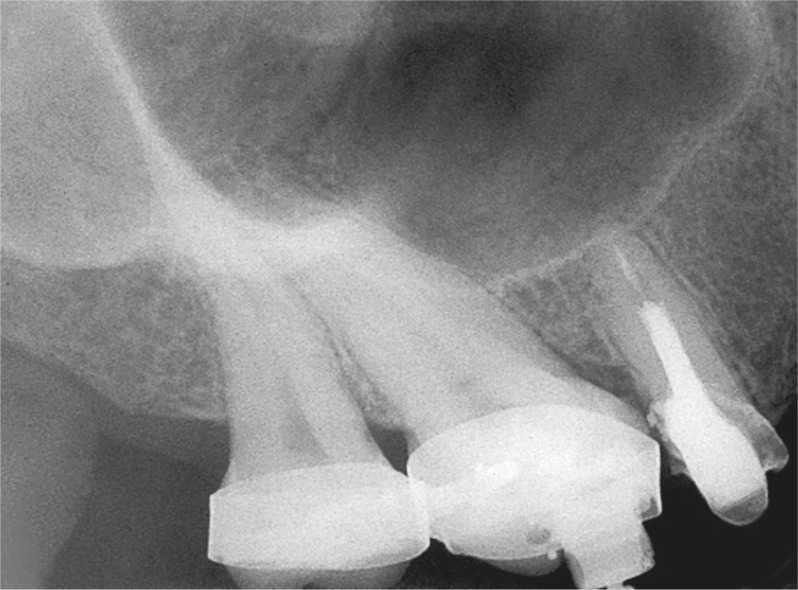
Mini-implant placement between #16 and 17 hindered by reduced space between roots.

**Figure 2 -:**
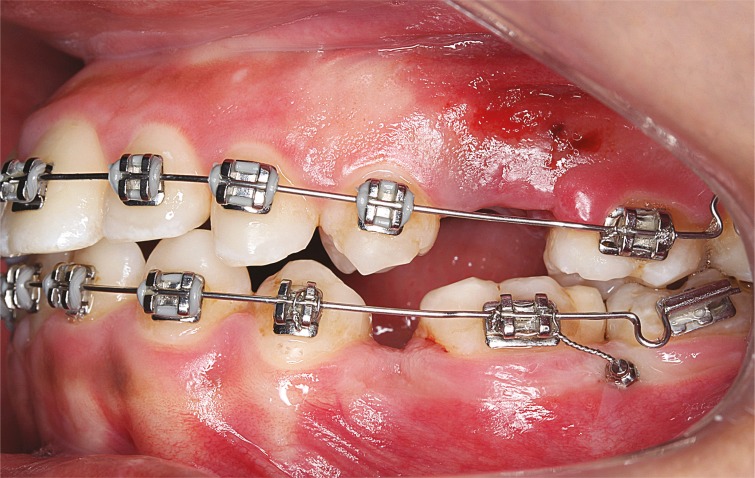
Implant placement failure in area of recent extraction.

Mini-implant placement site is suggested by the orthodontist who should elect it on the basis of the orthodontic mechanics of choice, distance between roots, attached gingiva dimensions, maxillary sinus height, magnitude of force, and bone density.^4^ In some cases, the site of professional choice is not the most appropriate for mini-implant placement (Fig 1). Certain regions in the oral cavity, such as the retromolar fossa, maxillary tuberosity and edentulous regions, present alveolar bone of questionable quality, which might lead to inefficient placement and mini-implant loss.^2 ^It is known that the retromolar fossa has buccal and lingual alveolar bone with favorable density; however, bone found at the center of this anatomical structure is porous, with large medullary spaces that hinder interlocking necessary for mini-implant stability.^2^ Similar bone feature is also found in the maxillary tuberosity and edentulous regions. It is worth noting that temporary anchorage devices (TADs) should not be placed in areas of recent extraction. A minimal 6-month interval should have passed in order to assure mature bone formation (Fig 2).

**Figure 3 -:**
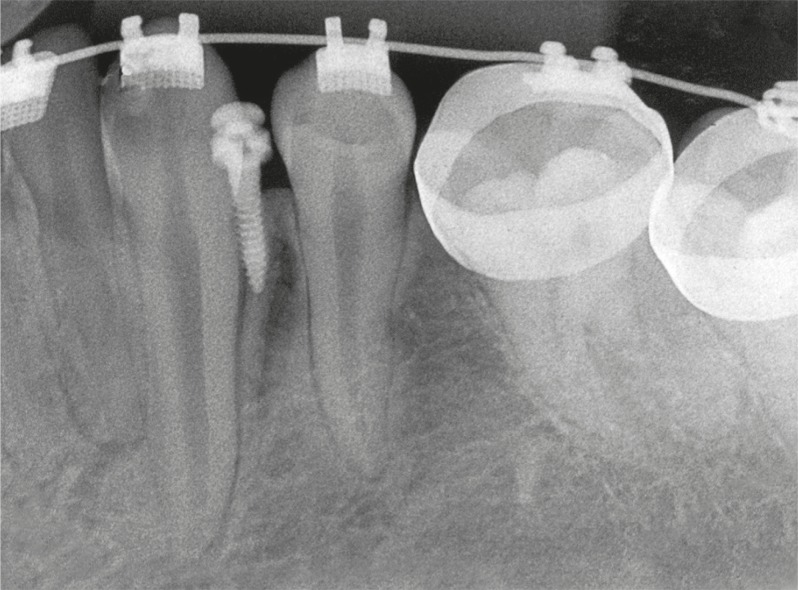
Mini-implant near the tooth root.

One disadvantage of mini-implants is that, most of times, they are placed between tooth roots, which increases the risk of damage and loss of stability.^11^ Placing a mini-implant over a tooth root might lead to cement destruction or TAD fracture. In less severe cases, mini-implants are placed near the root, which hinders thread placement in the alveolar bone, as part of a thread is inserted into the periodontal ligament (Fig 3). In this context, most of times, mini-implants present primary stability, but might be subject to loss after a few days or weeks due to vertical tooth movement.

**Figure 4 -:**
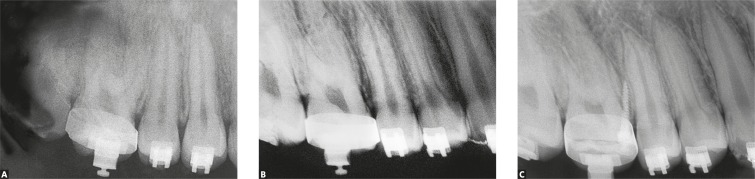
Periapical radiographs. A) Reduced space between #15 and 16 roots; B) Enlargement of space between roots after orthodontic bends were performed in the arch; C) Mini-implant placement.

Whenever radiographic examination, ridge palpation or root contour reveal that tooth roots are too near, specific orthodontic preparation should be carried out in order to upright the teeth and split roots apart (Fig 4). For a safe implant placement procedure, it is recommended that the space between roots respect mini-implant diameter plus 1 mm mesially and 1 mm distally. For instance: Should the mini-implant of choice be 1.5 mm in diameter, the space between roots should be of 3.5 mm.

**Figure 5 -:**
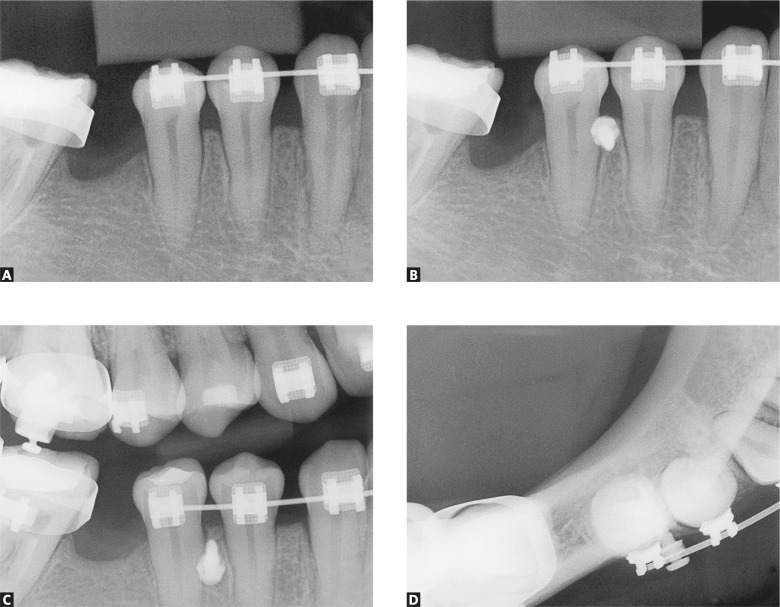
A) Initial periapical radiograph; B) Periapical radiograph after implant placement; C) Bitewing radiograph; D) Conventional occlusal radiograph.

It is advisable to avoid sites with insufficient amount of alveolar bone and decreased space between roots. Interproximal, periapical and even conventional occlusal radiographs might contribute to achieve greater success with the mini-implant placement technique and, therefore, increase stability (Fig 5). 

## 2) Different sites require mini-implants of different sizes! Implant size should respect alveolar bone diameter!

The ideal mini-implant should be as big as possible in order to assure greater stability. However, mini-implant length is determined by the buccolingual thickness of the alveolar bone in which it is inserted and by the presence of some important anatomical structures. Manufacturers suggest the ideal mini-implant size for each site, considering an additional safety margin, so as to achieve the required implant attachment. Longer (from 8 to 9 mm) devices are recommended in the upper posterior buccal region; whereas shorter ones (from 6 to 7 mm) are recommended in the upper anterior buccal region and lower arch, since, at the latter, alveolar bone is not as thick in buccolingual direction. Using mini-implants proper in size not only prevent perforations on the opposite side of the cortex, but also avoid poor thread placement into the alveolar bone.

Mini-implant diameter should also be considered. In general, larger diameters assure better primary stability, but might affect secondary stability.^9^ Kim et al^5 ^assessed the influence of shape over mini-implant stability. The authors state that cone-shaped mini-implants greater in diameter might cause excessive compression over the cortical bone, since they require greater insertion torque. Damages caused to the cortical bone might lead to ischemia, necrosis, bone remodeling and mobility, all of which might lead to mini-implant loss.^13^

It is recommended that mini-implants be 1.5 mm in diameter in upper and lower, anterior and posterior buccal sites. As for edentulous regions, maxillary tuberosity, retromolar fossa and mid palatal suture, 2 mm is recommended. Finally, for the palatal region, mini-implants should be 1.8 mm in diameter.

Care should also be taken when choosing the transmucosal profile according to the implant placement site, as it is necessary to ensure that the thread be inserted into the alveolar bone while the transmucosal profile be covered by gingival tissue. Profile usually varies from 0 to 3 mm and might be determined by an anaesthetic needle and/or a spear tip. For upper and lower buccal regions as well as the mid palatal suture, smaller profiles are recommended (from 0.5 to 1 mm); whereas the retromolar fossa and palatal/tuberosity regions require larger profiles (from 2 to 3 mm). Should the transmucosal profile be too small, it may lead to ischemia due to pressure exerted to the platform. Conversely, should it be too large, it causes discomfort to patients, as its head remains out of the gingival tissue.^9 ^The transmucosal profile should not interfere in mini-implants placement into the bone. 

## 3) Properly determining mini-implant placement site provides ease for implant placement and contributes to stability!

Prior to lancing procedures, additional care should be taken in order to ensure that all procedures be carried out without causing discomfort to patients, in addition to providing implant placement with a clinical sequence that provides ease for further steps. Determining the site of TAD placement is an important step for the surgical technique. A periodontal probe moving from mesial to distal allows identification of root positioning. A cavity is normally found between roots and that is where a mini-implant should be placed, as apical as possible and into the attached gingiva where alveolar bone is more compact, thus providing greater stability.^2^ Avoiding placing a mini-implant in free gingiva prevents the soft tissue from moving over the mini-implant, thus decreasing the risk of trauma. Procedures such as the application of topical anesthesia followed by infiltrative anesthesia allow lancing procedures to be carried out and favor mini-implant placement in gingival tissue, thereby reducing patient's discomfort. Future lancing procedures followed by mini-implant placement in bone tissue should be performed at the same site where infiltrative anesthesia was applied, which ensures that clinical sequence be strictly respected. Properly determining mini-implant placement site should occur during the primary procedures. Absence of discomfort favors final procedures.

## 4) The more precise the lancing procedures, the better the implant placement technique!

In the sequence of mini-implant placement technique, lancing procedures play an important role. The better the lancing procedures, the easier the path followed by a mini-implant inside the bone. Large spear tips macerate the gingival tissue, cause bleeding and subject the area to infection.

Lancing is considered a pre-implant placement procedure, as it should be carried out at the same site and with equal inclination of the mini-implant thread into the bone. It requires mild pressure manually applied by the clinician until the active spear tip enters the gingival tissue and the alveolar bone inner layer. Whenever the clinician feels a vacuum-like sensation, it means the procedure is concluded. Lancing procedures function as a guide, the initial perforation for future TAD interlock. 

Large spear tips, excess pressure and movement might lead to necrosis and microfracture in the alveolar bone, hindering mini-implant placement. It is paramount that clinical signs be observed. Difficulty in performing perforations might be a sign of inadequate position or contact with tooth root. On the other hand, easy perforations might be a sign of immature or insufficient alveolar bone. 

## 5) Self-drilling does not mean higher pressure!

Self-drilling mini-implants dispense prior drill perforation. For this reason, they are pressed over and manually thread into the alveolar bone based on the opening created by the spear tip. The thinnest mini-implant end is placed into the cavity while light pressure is applied. Subsequently, the device is thread by rotating the key hand, preferably without pressure. Should primary procedures be successfully performed, TAD will be easily thread with no need for manual force.

Nevertheless, proper placement implies pressure and, for this reason, clinicians often make the mistake of applying excess pressure over the mini-implant during placement. This may displace a mini-implant out of its initial position and lead to microfractures and alveolar bone enlargement, thereby weakening mini-implant interlocking and leading to premature absolute anchorage loss.

Insertion torque is directly related to mini-implant size, bone density and professional experience, and directly influences stability.^3^^,^ 9 It should range from 5 to 10 N/cm^2^ for devices 1.5 mm in diameter and 20N for thicker mini-implants (2.0 mm). Current keys in use have a torque gauge that allows the operator to control torque.^8^

After lancing procedures and mild primary pressure, rotating movements should be applied with the aid of a key hand, which will contribute to the success rate of mini-implants.

## 6) Knowing where implant placement should end decreases the risk of complications and mini-implant loss!

Properly ending a procedure is just as important as starting well, and is a goal to be achieved. It is common knowledge within the orthodontic scientific community that a mini-implant comprises three parts: head, transmucosal profile and active tip (thread). The active tip should be completely inserted into the bone while the transmucosal profile should be totally covered by tissue. The head functions as a connecting link between the TAD and the orthodontic mechanics, and should be passively supported by the gingival tissue, that is, outside the gingiva but in direct contact with it. Thus, mini-implant should be thread so as to achieve such position. For this to occur as natural as possible, all aforementioned steps should have been strictly followed, from adequate mini-implant to proper placement site.^2^

Should excess pressure be applied to the platform of the mini-implant head, gingival tissue becomes ischemic and this condition will not cease overtime. Should that be the case, gingival tissue responds with inflammation and, in more severe situations, necrosis. Furthermore, the patient might feel pain, which hinders hygiene around the device and, thus, leads to mucositis or peri-implantitis. All the aforementioned situations can contribute to early mini-implant loss. 

It is advisable to thread the mini-implant once in a while and periodically assess how near it is in relation to patient's gingiva, so as to achieve ideal implant placement.

Pressing the mini-implant head over gingival tissue does not increase thread interlocking into the alveolar bone, instead, it damages the periodontium and hinders stability. Major steps for TAD placement are shown inFigure 6.

**Figure 6 -:**
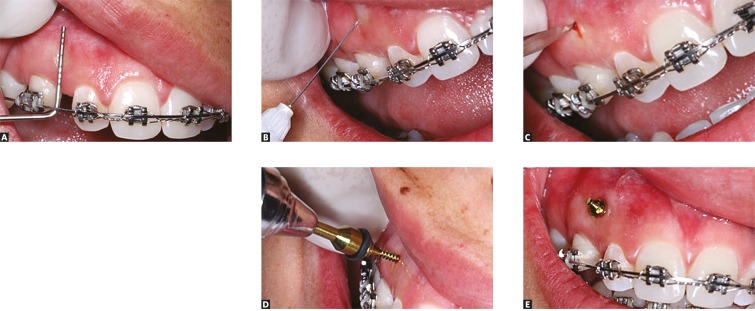
Major steps for mini-implant placement. A) Choosing ideal mini-implant placement site with the aid of a millimeter periodontal probe; B) Infiltrative anesthesia; C) Lancing procedures; D) Implant placement onset; E) Mini-implant in place. (Images provided by Prof. Dr. Antônio Carlos Ruellas).

## Final considerations

Mini-implant loss might be associated with the implant placement technique!

It is worth highlighting that:

1) In some cases, the site of professional choice is not the most appropriate for mini-implant placement. Alveolar bone of questionable quality and reduced space between roots should be avoided.
2) Mini-implant length is determined by the buccolingual thickness of the alveolar bone. Large diameters weaken the alveolar bone. On the other hand, bicortical mini-implant anchorage increases stability.
3) Clinical examination, assessment of root contour and CT scans decrease the risk of flaws.
4) Precise lancing procedures and pressure firm enough to perforate the alveolar bone provide ease for mini-implant threading.
5) After primary interlocking, no pressure should be applied over mini-implants.
6) A mini-implant should be placed with the thread inside the alveolar bone, the transmucosal profile covered by gingival tissue and the head supported by the gingiva.
7) Mini-implant placement should be performed after careful planning and by means of a judicious technique.

## References

[1] Araújo TM, Nascimento MHA, Bezerra F, Sobral MC (2006). Ancoragem esquelética em Ortodontia com
miniimplantes. Rev Dental Press Ortod Ortop Facial.

[2] Consolaro A, Romano FL (2014). Reasons for mini-implants failure: choosing installation
site should be valued!. Dental Press J Orthod.

[3] Buschang PH, Carrillo R, Ozenbaugh B, Rossouw PE (2008). 2008 survey of AAO members on miniscrew
usage. J Clin Orthod.

[4] Elias CN, Ruellas ACO, Marins EC (2011). Resistência mecânica e aplicações clínicas de
mini-implantes ortodônticos. Rev Bras Odontol;.

[5] Kim JW, Baek SH, Kim TW, Chang YI (2008). Comparison of stability between cylindrical and conical
type mini-implants: Mechanical and histological properties. Angle Orthod.

[6] Miyawaki S, Koyama I, Inoue M, Mishima K, Sugahara T, Takano-Yamamoto T (2003). Factors associated with the stability of titanium screws
placed in the posterior region for orthodontic anchorage. Am J Orthod Dentofacial Orthop.

[7] Reynders RA, Ronchi L, Bipat S (2009). Mini-implants in orthodontics: a systematic review of
the literature. Am J Orthod Dentofacial Orthop.

[8] Reynders RA, Ronchi L, Ladu L, van Etten-Jamaludin F, Bipat S (2012). Insertion torque and success of orthodontic
mini-implants: a systematic review. Am J Orthod Dentofacial Orthop.

[9] Ruellas ACO, Ruellas ACO (2013). Mini-implantes. Biomecânica aplicada à clínica.

[10] Ruellas ACO, Mattos CT, Elias CN (2012). Avaliação dos torques de inserção e remoção e da
resistência mecânica de novos mini-implantes ortodônticos. Orthod Science Pract.

[11] Shinohara A, Motoyoshi M, Uchida Y, Shimizu N (2013). Root proximity and inclination of orthodontic
mini-implants after placement: cone beam computed tomography
evalutation. Am J Orthod Dentofacial Orthop.

[12] Takaki T, Tamura N, Yamamoto M, Takano N, Shibahara T, Yasumura T (2010). Clinical study of temporary anchorage devices for
orthodontic treatment-stability of micro/mini-screws and mini-plates: experience
with 455 cases. Bull Tokyo Dent Coll.

[13] Wawrzinek C, Sommer T, Fischer-Brandies H (2008). Microdamage in cortical bone due to the overtightening
of orthodontic microscrews. J Orofac Orthop.

[14] Wiechmann D, Meyer U, Buchter A (2007). Sucess rate mini- and micro-implants used for
orthodontic anchorage: a prospective clinical study. Clin Oral Implants Res.

